# Therapeutic Potential of Salvinorin A and Its Analogues in Various Neurological Disorders

**Published:** 2022-06-29

**Authors:** Joseph Cichon, Renyu Liu, Hoang V. Le

**Affiliations:** 1 Department of Anesthesiology and Critical Care, Perelman School of Medicine, University of Pennsylvania, Philadelphia, PA, USA; 2 Department of BioMolecular Sciences and Research Institute of Pharmaceutical Sciences, School of Pharmacy, University of Mississippi, University, MS, USA

## Introduction

**Figure 1:**
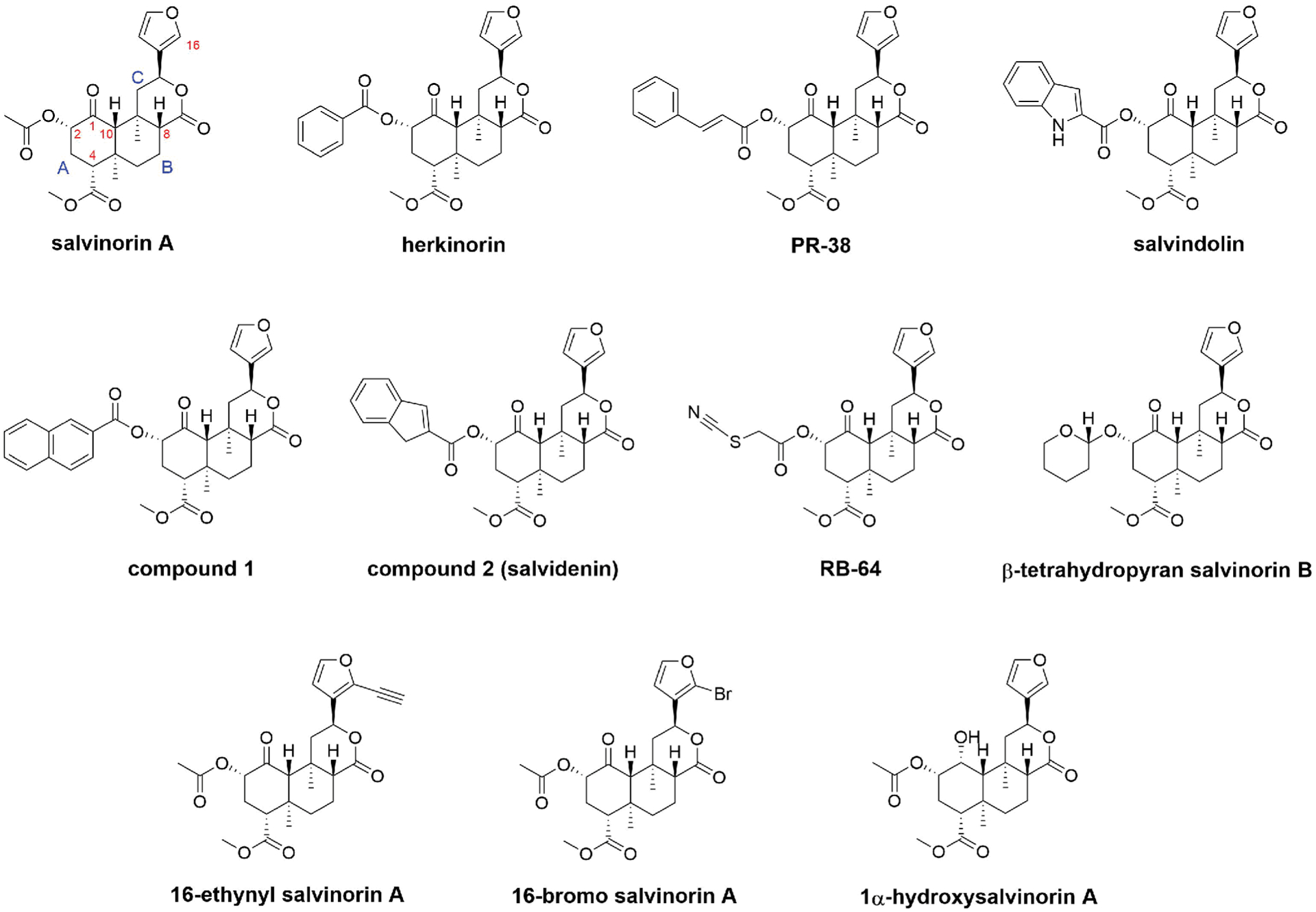
Chemical structures of salvinorin A and some well-known analogues of salvinorin A. Herkinorin, PR-38, salvindolin, compound 1, and compound 2 (now known as salvidenin) are dual KOR/MOR agonists. RB-64 is a G-protein-biased KOR agonist that forms a covalent bond with KOR. β-Tetrahydropyran salvinorin B is a long-acting KOR agonist. 16-Ethynyl salvinorin A is a nonbiased KOR agonist. 16-Bromo salvinorin A is a G-protein-biased KOR agonist. 1α-Hydroxysalvinorin A is a KOR antagonist. KOR, kappa opioid receptor. MOR, mu opioid receptor.

While the hallucinogenic plant *Salvia divinorum* has been safely consumed by humans for centuries for religious or recreational purposes without long-term side effects, its active component, salvinorin A, and molecular mechanism of action were not known until recently [1]. Notably, salvinorin A is a potent kappa opioid receptor (KOR) agonist that is highly selective for KOR without significant interactions with any other receptors [2]. Different from classic opioid receptor ligands, which are opiate, this is the first molecule that has no nitrogen in its chemical structure but can interact with an opioid receptor with high affinity and potency (Figure 1) [1]. All classic opioid receptor agonists or antagonists have nitrogen in their chemical structures; therefore, it had been hypothesized that nitrogen was essential for a compound to have any opioid receptor activity until salvinorin A was discovered. In addition to its unique chemical structure, salvinorin A has very high selectivity towards KOR. Thus, it has been used as a great tool to study the pure pharmacological effects of KOR agonism. The C2 ester in the chemical structure quickly undergoes hydrolysis to yield the inactive metabolite salvinorin B, resulting in salvinorin A undergoing fast metabolism in the body. Despite its short duration of action, a single dose of salvinorin A, like other classic psychedelics, induces rapid and robust changes in the patterns of neural activity and connectivity in disease-related neuronal circuits. While not fully understood, these neurophysiological effects might explain salvinorin A’s rapid and durable therapeutic effects in neuropsychiatric disorders that are difficult to treat. Furthermore, the unique chemical structure of salvinorin A provides a useful backbone for chemists to develop novel compounds targeting KOR and other opioid receptors for structure-activity relationships, structure-function analyses, and novel medication design purposes [3,4].

## Chemical Properties of Salvinorin A

Salvinorin A is a *neo*-clerodane diterpenoid. It is not soluble in water or lipids, which is a significant hurdle for clinical deliverable formulation. In contrast to classic KOR ligands, salvinorin A is not an alkaloid, indicating that it cannot be rendered into a salt to improve solubility for drug development purposes. The melting point of salvinorin A is high (238–240 °C) [5], and the powder form of salvinorin A is relatively stable. However, once taken, salvinorin A is hydrolyzed quickly by esterases due to the hydrolyzable nature of the ester functional group at C2, which is an advantage for clinical practices that need the benefits of a KOR agonist with a short-acting property and a short duration of hallucinatory or dissociative side effects (~10–15 minutes).

## Molecular Target of Salvinorin A

Salvinorin A (Figure 1) was first isolated from *Salvia divinorum* in 1982 by Ortega and coworkers [6], and it is one of the most potent, naturally occurring opioid agonists, with high selectivity and affinity towards KOR (*K*_i_ = 4 nM, *EC*_50_ in [^35^S]GTPγS binding assay = 2.2 nM) [7]. In 2002, Roth and coworkers discovered that salvinorin A targeted KORs expressed in both human embryonic kidney-293 cells (*K*_i_ = 16 nM) and guinea pig brain (*K*_i_ = 4.3 nM) [1]. They also discovered the nonbiased agonism nature of salvinorin A towards KOR when it was observed to activate both the G-protein signaling pathway (*EC*_50_ in cAMP assay = 4.73 nM) and β-arrestin-recruitment pathway (*EC*_50_ in the Tango assay = 10.5 nM) [8].

## Analogues of Salvinorin A

Many analogues of salvinorin A have been developed to target different opioid receptors [4,7]. The most common alteration to the structure of salvinorin A is the replacement of the acetate at C2. Many different functional groups at this position, including carbonates, carbamates, alternative ester groups, ethers, amines, amides, sulfonic esters, sulfonamides, thioesters, and halides, have been incorporated and studied [4]. Data from these studies have suggested that the C2 position is critical for KOR binding and activation [7]. Notably, several C2 esters with a conjugated ring, an aromatic ring, or fused rings, such as herkinorin [9], PR-38 [10], salvindolin [11], compound 1 [12], and compound **2** (now known as salvidenin) [3] (Figure 1), displayed dual agonism on KOR and mu opioid receptor (MOR) [3]. Dual KOR/MOR agonists have been shown to retain analgesic activity while showing reduced undesirable adverse effects compared to pure KOR agonists or pure MOR agonists [3]. For example, male C57BL/6NHsd mice treated with salvidenin showed a significant increase in the latency to paw response in a hot plate test (single dose 2 mg/kg, i.p.) compared to vehicle-treated mice, which indicated antinociception, and showed a significant increase in the amount of time spent on the open arms in an elevated plus maze test (single dose 5 mg/kg, i.p.), which indicated anxiolysis [3]. Another notable C2 ester is RB-64 (Figure 1), which is the first and only known salvinorin-based agonist that forms a covalent bond with KOR [8]. RB-64 is also known to be G-protein biased. It displayed functional selectivity for G-protein over β-arrestin recruitment by a factor of 96 [8]. Therefore, RB-64, along with salvinorin A, have been widely used to study the specific effects and differences between G-protein signaling and β-arrestin recruitment signaling.

Other alternations to the structure of salvinorin A, such as the replacement of the methyl ester at C4, modifications of substituents on rings A and C, and replacement of the furan ring, have also been prepared and evaluated [4]. Unlike the acetate group at C2, the methyl ester at C4 requires more forcing conditions for hydrolysis and modification. So far, all of the modifications at the C4 position have resulted in a loss of binding affinity and potency on KOR [4]. Similarly, so far, none of the modifications of substituents on rings A and C or replacement of the furan ring have resulted in compounds with improved binding affinity and potency on KOR compared to salvinorin A [4,7]. Recently, substitution on the furan ring at C16 led to two interesting salvinorin A analogues, 16-ethynyl salvinorin A and 16-bromo salvinorin A (Figure 1) [13]. While 16-ethynyl salvinorin A displayed balanced signaling properties, 16-bromo salvinorin A showed a significant G-protein signaling bias. In acute nociceptive and inflammatory pain mouse models, 16-ethynyl salvinorin A showed significant antinociceptive effects and reduced side effects compared to salvinorin A, while 16-bromo salvinorin A displayed modest antinociceptive effects and lacked anxiogenic effects [13].

Interestingly, some small modifications to the salvinorin structure at C1 or C10 were observed to switch the functionality of the molecule from an agonist to an antagonist of KOR [14]. There are only six salvinorin-based compounds in the literature that have demonstrated antagonistic activity against any of the opioid receptors; 1α-hydroxysalvinorin A (Figure 1) is the most potent and selective antagonist for KOR [3]. KOR antagonists have been shown to alleviate depressive and anxiety-related disorders, which are the common issues related to withdrawal that can lead to drug relapse. Recently, 1α-hydroxysalvinorin A and nor-BNI were studied on C57BL/6N mice for spontaneous cocaine withdrawal [15]. Administration of 1α-hydroxysalvinorin A (5 mg/kg, i.p.) was shown to reduce spontaneous cocaine-withdrawal behaviors, comparable to nor-BNI (5 mg/kg, i.p.). Notably, 1α-hydroxysalvinorin A produced anti-anxiety-like effects in the light-dark transition test that was not observed with nor-BNI (both 5 mg/kg, i.p.). Still, the mechanisms of action of KOR antagonists on withdrawal have not been known.

## Salvinorin A and Neuronal Circuits Modulation

**Figure 2:**
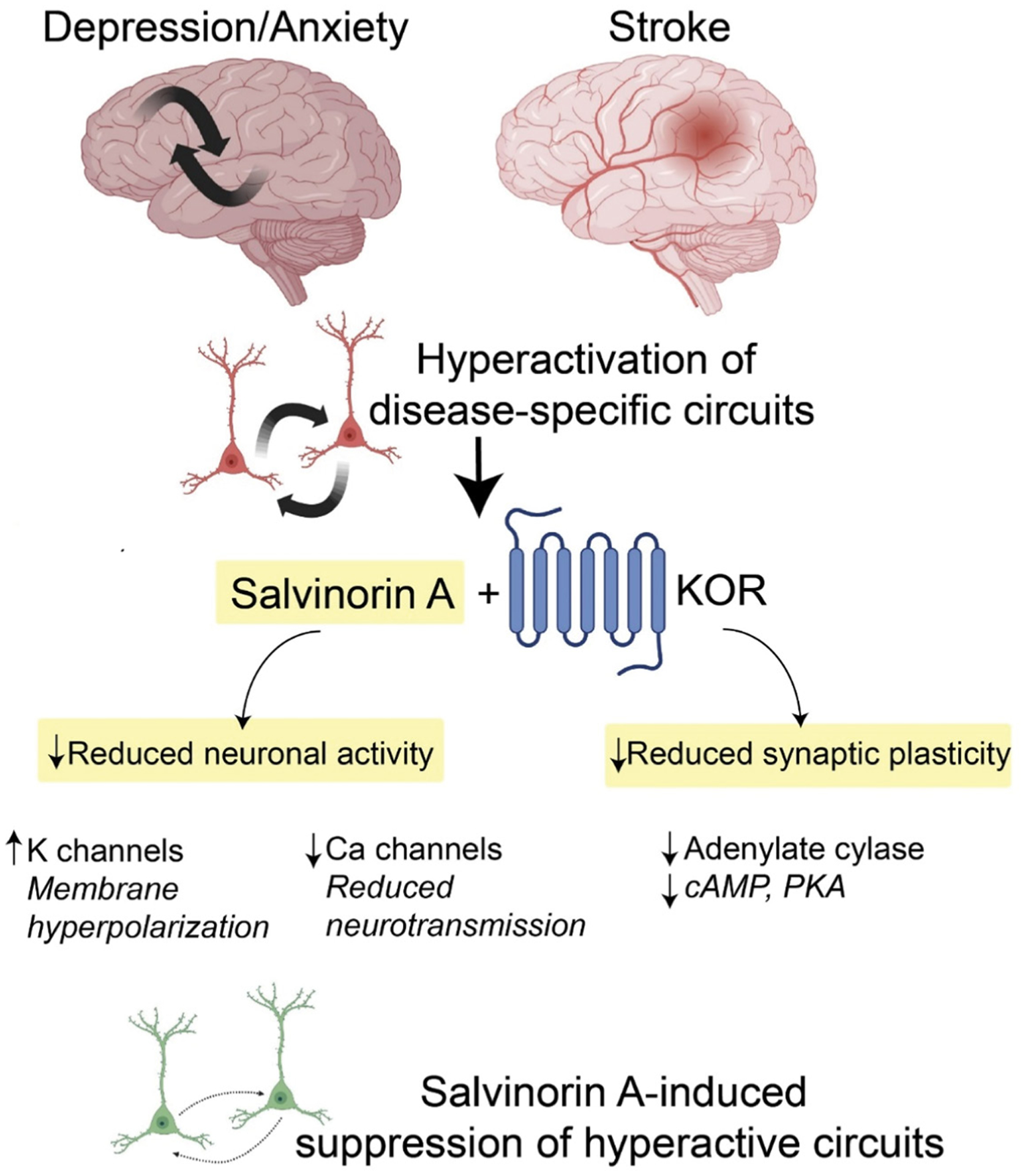
Schematic showing the potential of salvinorin A in modulating neuronal activity and plasticity in the brain. Certain disease states could drive hyperexcitity in distinct circuits. Delivery of salvinorin A and KOR activation can rapidly decrease neuronal activity and synaptic plasticity. These rapid and sustained effects might reshape the connectivity and patterns of neuronal activity towards normal brain function. KOR, kappa opioid receptor. K, potassium. Ca, calcium. cAMP, cyclic adenosine monophosphate. PKA, protein kinase A.

The psychoactive effects of salvinorin A have in large part hindered its development and characterization as a therapeutic agent in modern medicine. The salvinorin A-induced experience is hallmarked by drastic perceptual changes, hallucination (visions and auditory), intense feelings of depersonalization and derealization, and dissociative effects (unresponsiveness to environmental stimuli) [16]. In rodents and non-human primates, salvinorin A causes a degree of sedation and impaired locomotion [17]. These experiences have been sought out by Mazatec shamans for medical and religious purposes since neolithic times [18]. Interestingly, only until recently has there been a growing appreciation of previously known psychedelic substances, including salvinorin A, in the treatment of complex human brain disorders and chronic disease states. Increasing evidence suggests these agents induce rapid and robust therapeutic effects in select patient populations who suffer from anxiety, chronic stress, depression/mood disorders, substance disorders, and pain conditions [19]. While most of these agents target serotonin 2A receptor (psilocybin and lysergic acid diethylamide (LSD)) or N-methyl-D-aspartate (NMDA) receptor (ketamine and nitrous oxide), salvinorin A mediates its effects through KOR activation. KOR couples primarily to G*i/o* proteins that regulate intracellular IP3- and cAMP3-based second messenger cascades. Activation of this signaling pathway decreases cellular excitability via an increase of the inward rectifier potassium currents [20]. Additionally, KOR down-regulates N-type calcium currents, which, via the reduction of presynaptic calcium influx, likely reduces the release of both excitatory and inhibitory neurotransmitters. Collectively, KOR activation would be inhibitory by reducing both the input signals and the postsynaptic responses. The neurophysiological effects of salvinorin A *in vivo* and in the treatment of various neurological disorders are not known. However, based on KOR’s expression profile, including the striatum, several cortical areas (deep cortical layers (V)), limbic areas (hippocampus and amygdala), hypothalamus, spinal cord, and the claustrum, salvinorin A has been hypothesized to downregulate the neuronal activity in distinct circuits tied to the aforementioned areas [21]. Thus, salvinorin A might be well suited to acutely inhibit subsets of hyperactive neurons to resculpt abnormal patterns of neuronal activity, potentially engage various forms of synaptic plasticity, and normalize brain function (Figure 2). In ischemic stroke, for example, abnormal glutamate release drives hyperexcitability and brain injury and worsens functional outcomes. Administration of salvinorin A to patients early in stroke evolution might suppress glutamate-induced hyperactivation to minimize brain injury. This hypothesis could be carried over to treat neuropsychiatric diseases characterized by circuits with motifs of hyperactivation; for example, anxiety disorders and post-traumatic stress disorders have been linked to cortico-amygdala hyperactivation where salvinorin A might provide specific suppression of these activity patterns and the grounds for weakening such connections.

## Potential Clinical Implications

Salvinorin A has therapeutic potential in various complex diseases and conditions including pain [22], addiction [23], depression [24], itching [23], and stroke [25]. In general, salvinorin A’s short-acting activity has hindered its clinical utility since most of the aforementioned clinical situations prefer relatively long-acting compounds. Some success has been made to extend its half-life by replacing the ester at C2 with more stable functional groups, such as carbamates, ethers, and amides. A recent study showedthat β-tetrahydropyran salvinorin B (Figure 1), a C2-ether analogue of salvinorin A, had a longer duration of action and displayed analgesic and anti-inflammatory effects in mice [26]. Our group has been focusing on the application of salvinorin A in acute stroke by taking advantage of its high potency and rapid onset. The effectiveness of salvinorin A in acute stroke rescue has been demonstrated in multiple animal models, including rodents [27,28], piglets [29,30], and monkeys [25]. The short-lived dissociative effects of salvinorin A have been considered by some as a hard stop in this patient population. However, it is possible that the unwanted effects might actually be therapeutic for neuronal circuit function and brain health. Salvinorin A’s rapid change in neuronal activity and potential for synaptic plasticity in the brain might set the stage for the beneficial effects that are essential for stroke recovery. Additionally, in some patients where psychoactive effects might be deemed intolerable, molecular modifications of salvinorin A to add dual MOR activation could be reasonable [3]. A summary of the potential clinical usages of salvinorin A and its analogues is presented in Table 1.

## Figures and Tables

**Table 1:** Therapeutic potentials of salvinorin A and its analogues.

Compound	Therapeutic Potential
Salvinorin A	Analgesic [31], addiction therapy [32], anti-cocaine seeking therapy [33], antidepressant [32], antipruritic [34], stroke rescue [25,28,35]
Salvindolin	Antidepressant [11], analgesic [11]
PR-38	Colitis [36], irritable bowel syndrome [37], antipruritic [38]
1α-Hydroxysalvinorin A	Anxiolytic [15], cocaine withdrawal therapy [15]
RB-64	Analgesic [8]
Salvidenin	Analgesic [3], anxiolytic [3]
16-Ethynyl salvinorin A	Analgesic [13], attenuation of cocaine-induced reinstatement [39]
16-Bromo salvinorin A	Analgesic [13], attenuation of cocaine-induced reinstatement [39]
β-Tetrahydropyran salvinorin B	Analgesic [26], anti-inflammatory [26], cocaine withdrawal therapy [40], addiction therapy [40]
Herkinorin	Anti-inflammatory [41], stroke rescue [42]
